# The role of inflammatory markers following Ramadan Fasting

**DOI:** 10.12669/pjms.35.1.95

**Published:** 2019

**Authors:** Rubina Mushtaq, Ambreen Akram, Rehana Mushtaq, Sobia Khwaja, Shabbir Ahmed

**Affiliations:** 1 Rubina Mushtaq, Post Doc. Professor and Dean Faculty of Science, Department of Zoology, Federal Urdu University of Arts Science and Technology, Gulshan e Iqbal Campus, Karachi, Pakistan; 2 Ambreen Akram, PhD. Assistant Professor, Department of Zoology, Federal Urdu University of Arts Science and Technology, Gulshan e Iqbal Campus, Karachi, Pakistan; 3 Rehana Mushtaq, PhD. HOD and Professor, Department of Zoology, University of Baluchistan, Quetta, Pakistan; 4 Sobia Khwaja, PhD. Assistant Professor, Department of Zoology, Federal Urdu University of Arts Science and Technology, Gulshan e Iqbal Campus, Karachi, Pakistan; 5 Shabbir Ahmed PhD. Research Scholars, Department of Zoology, Federal Urdu University of Arts Science and Technology, Gulshan e Iqbal Campus, Karachi, Pakistan

**Keywords:** Ramadan Fasting, BMI, Adiponectin, TNF-α

## Abstract

**Objectives:**

The aim of present study was to investigate the effect of fasting during Ramadan on plasma adiponectin and TNF-α levels.

**Methods:**

This is a cross sectional study, conducted at Federal Urdu University of Arts, Science & Technology (FUUAST), Karachi, comprising a total of 55 (50%) females and 55 (50%) males whose ages ranged between 20 and 40 years, and fasted during Ramadan (June-July 2014) were enrolled in the study. Subjects were separated into normal weight, overweight and obese males and females. Anthropometric measurements and Fasting venous blood samples were taken at first and last (29^th^) day of Ramadan. Plasma adiponectin and TNF-alpha levels were assayed with ELISA kits. All values were calculated and presented as mean ± standard error of the mean (SEM) and by using analysis of variance (ANOVA) for repeated measures. P values < 0.05 were accepted as significant.

**Results:**

Body mass index (BMI) (Kg/m2) in over-weight and obese male subjects exhibited considerable reduction (P<0.05; P<0.05), post Ramadan when compared to their respective pre Ramadan fasting weights. Noticeable and significant reduction was also observed in BMI of obese females (P<0.05). Post Ramadan Overweight Males (P<0.05) and Post Ramadan Obese Males (P<0.001) exhibited significantly elevated plasma adiponectin (μg/mL) values. While plasma adiponectin mean concentration of only obese females were significantly improved at last week of Ramadan (P<0.01). Fasting in Ramadan significantly decreased TNF-α (pg/mL) levels of post obese males and females than Pre-Ramadan-groups (P<0.05; P<0.01) respectively.

**Conclusion:**

The study reports of noticeable changes with Ramadan fasting resulting increase of plasma adiponectin and decrease of TNF-α levels as well as body weight. The study strongly suggests further investigations on larger sample sizes with possible association of dietary restrictions and weight loss on mechanism of enhanced adiponectin and reduced TNF-α in obese and overweight persons who fast on Ramadan pattern.

## INTRODUCTION

Adipocytes create cytokines and adipocytokines with basic administrative consequences for inflammation, insulin sensitivity, coagulation, vascular homeostasis, hunger, energy, and so on. Disturbing influence in these administrative impacts prompts to insulin resistance and cardiovascular diseases.^1^ Ramadan fasting is associated with alteration in classically activated macrophage regulation/signaling and increase macrophage function and of pro-inflammatory cytokines and immune cells in healthy subjects.^2^,^3^ Adiponectin with its immune function and anti-inflammatory action can help this beneficial balance of cytokines during the fasting.^4^

Adiponectin improving insulin capacity and creating anti-atherogenic and anti-inflammatory impacts,^5^ this hormone regulates blood glucose level.^6^ Plasma level of adiponectin reduces in obesity, diabetes, in patients with metabolic disorder and cardiovascular issues.^5^ Low plasma adiponectin concentration is an alarming situation for cardiovascular illnesses.^7^ According to various epidemiological studies Plasma adiponectin level is inversely related to adipose tissue mass.^8^

Fasting helps in therapeutic treatment for different health issues including weight control.^9^ A consistent dietary limitation could decidedly impact the biochemical and physiological processes and the provocative condition of the body.^10^ Inflammatory status of the body adds to the pathogenesis of some important issue, for example, atherosclerosis, Insulin resistance, cardiovascular sicknesses and cancers.^11^

Aksungar et al. reported that inflammatory biomarkers had a huge diminishment after Ramadan in both the sexes contrasted with a week before Ramadan.^12^ Kacimi et al. expressed that there is a huge reduction in the circulatory level of inflammatory cytokines during Ramadan fasting.^2^ Unalacak et al. found that TNF-α diminished during fasting.^10^ Moreover, it is trusted that these cytokines repress Lipoprotein Lipase (LPL) action prompting to the down-control of inflammation in fasting people.^13^

Feizollahzadeh et al. examined that Ramadan fasting improves human health. As indicated by the outcomes, fasting in Ramadan altogether improved increment in serum adiponectin concentrations of patients with Type II diabetes.^14^ Adiponectin play a regulatory role in the incidence of insulin resistance.^15^

Present study was planned to investigate the effect of fasting during Ramadan on plasma adiponectin and TNF-α levels in obese, overweight and normal weight people.

## METHODS

This study was carried out in June - July 2014 during the month of holy Ramadan, The Research Ethics Committee of Federal Urdu University of Arts Science and Technology (FUUAST), Karachi approved study protocol. Participants for this study were recruited from FUUAST and some other localities of Karachi, Pakistan. Volunteers were approached a week before Ramadan and a written informed consent was obtained. Inclusion criteria for the study was all participants who were with normal weight, overweight and obese as well as fast throughout the month of Ramadan and are not suffering from any chronic disease. Pregnant women, subject with cardiovascular diseases and of age more than 40 years were excluded. A total of 55 female and 55 male volunteers, ages ranged between 20 and 40 years underwent anthropometric, and biochemical evaluation on first day and at the end of Ramadan. Subjects were grouped into three BMI categories i.e., control, overweight and obese. Among males, 15 (27.27%) normal weight, 10 (18.18%) overweight and 30 (54.54%) obese volunteers were included in this study. While among females, 15 (27.27%) normal weight, 10 (18.18%) overweight and 30 (54.54%) obese females participated.

All the subjects fasted on the pattern of and throughout the Ramadan resulting in average fasting time of about 15 hours a day. Females continued to fast on the pattern despite of their break during menstruation for the quality experiment. All the subjects were kept on dietary restrictions; all of them were suggested to avoid oily foods stuff at Iftar (breaking of fast time) as well as provided with white oats (bran diet) for Sahar (onset of fasting time) meal.

Anthropometric measurements like weight and height were taken at 1^st^ Ramadan and on the last sampling day of Ramadan then BMI was calculated. Similarly venous blood sample were taken on 1^st^ and 29^th^ day of Ramadan just before Iftar from each subject. Blood samples were processed and centrifuged for serum separation.

For Estimation of Adiponectin, Human Adiponectin (ADP) ELISA kit (Catalog # 95374 Glory Science Co., Ltd USA) and for TNF-α, Human TNF-α ELISA kit (Catalog # 950.090.096 Diaclone SAS, France) was used.

Statistical analysis was achieved using the SPSS statistical software (SPSS, Version 23). All values were calculated and presented as mean ± standard error of the mean (SEM) and by using analysis of variance (ANOVA) for repeated measures. P values < 0.05 were accepted as significant.

## RESULTS

**Fig.1:**
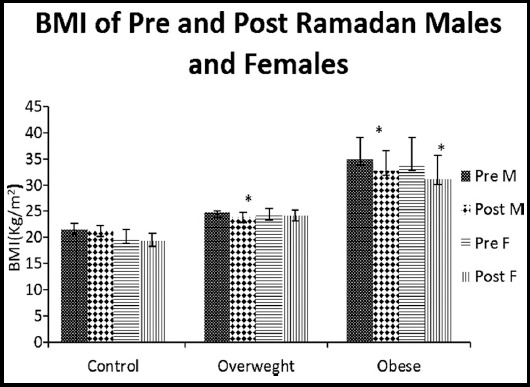
BMI (kg/m^2^) of Pre and Post Ramadan males and females.

Mean age of normal weight, overweight and obese male subjects was 24.73 ± 0.65, 32.10 ± 1.58, 33.90 ± 1.38 years respectively. BMI (Kg/m^2^) of the pre and post Ramadan normal weight males (21.56 ± 1.11; 21.25 ± 1.07), pre and post overweight (24.75 ± 0.33; 23.83±1.00) and pre and post obese males (34.90 ± 4.11; 32.78 ± 3.85). The post Ramadan value in over-weight and obese male subjects showed considerable reduction (P<0.05) when compared to their respective pre Ramadan value (Fig.1).

Average age of normal weight, overweight and obese females were 21.80 ± 0.44, 30.70 ± 1.42 and 31.83 ± 1.16 years respectively. BMI values of pre and post normal weight were (19.84 ± 1.73; 19.28 ± 1.50), pre and post overweight females were (24.36 ± 1.10; 24.13 ± 1.06) and pre and post Ramadan obese females were (33.80 ± 5.26; 31.06 ± 4.57) respectively. BMI of post-obese females was observed reduced considerably (P<0.05) (Fig.1).

**Fig.2:**
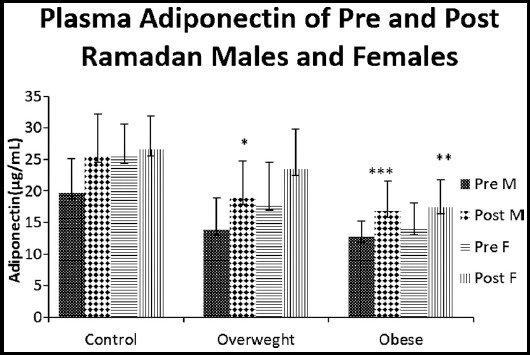
Adiponectin level (μg/mL) of Pre and Post Ramadan males and females

Plasma adiponectin level (μg/mL) of pre- and post Ramadan sample in normal weight males were 19.66 ± 5.47 versus 25.53 ± 6.65, respectively and in overweight males the respective values were 13.90 ± 5.04 and 18.90 ± 5.85. Average pre and post Ramadan sample plasma adiponectin levels were (12.76 ± 2.48 and 16.83 ± 4.68) in obese male subjects. Thus in overweight and obese males comparisons to their respective pre- levels post Ramadan sample showed significantly elevated (P<0.05) and (P<0.001) levels respectively (Fig.2).

Plasma adiponectin pre- and post Ramadan level (μg/mL) of normal weight females were 25.40 ± 5.19 and 26.60 ± 5.27 respectively, while those of overweight females were 18.00 ± 6.58 and 23.50 ± 6.32 respectively and of obese females were 14.06 ± 4.01 and 17.36 ± 4.46. Our findings determined that plasma adiponectin mean concentrations of obese females were significantly improved at the conclusion of the Ramadan (P<0.01) (Fig.2).

**Fig.3:**
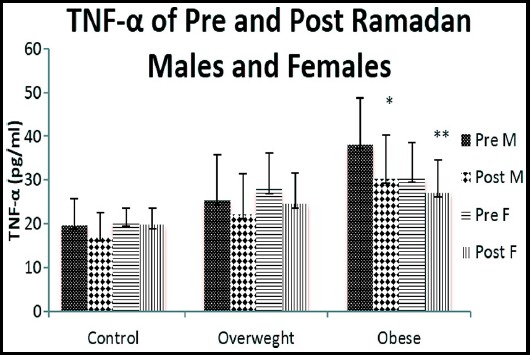
TNF-α (pg/ml) of Pre and Post Ramadan males and females.

Plasma TNF-α (pg/mL) level of pre- and post Ramadan samples in normal weight males were averaged at 19.66 ± 6.07and 17.13 ± 5.36 respectively. In overweight pre- and post Ramadan average TNF-α concentration were 25.36 ± 10.34 and 22.16 ± 9.30 pg/mL respectively. While in obese males were 36.08 ± 10.71 and 30.20 ± 10.17 pg/mL respectively. Our finding showed post Ramadan plasma TNF-α levels were significantly decreased than pre Ramadan (P<0.05) (Fig.3).

Plasma TNF-α level of pre Ramadan and post Ramadan normal weight females was 20.38 ± 3.21 and 19.86 ± 3.81 pg/mL respectively. Pre Ramadan and post Ramadan value in overweight females were measured at 27.97 ± 8.12 and 24.50 ± 7.24 pg/mL respectively, while pre Ramadan and post Ramadan obese females were 30.49 ± 7.98 and 27.17 ± 7.34 pg/mL respectively. Results of our experiment showed plasma TNF-α mean values of post Ramadan were reduced than that of pre Ramadan status and result was statistically significant (P<0.01) (Fig.3).

## DISCUSSION

Findings of current study demonstrated that weight and BMI significantly decreased among the subjects following Ramadan. The results of this study were in agreement with studies carried out by other authors.^16^ Weight loss during Ramadan may be associated with mild dehydration due to fasting.^17^ Previous literature showed a significant reduction in body weight regardless of no significant differences in energy intake. It could be assumed that weight loss during fasting period is to some extent due to effective decline of body fat mass.^18^

The present study revealed that Ramadan fasting was coupled with significant increase in plasma level of adiponectin and reduction in TNF alpha among obese men as well as women. Aksungar et al. also investigated the effects of Ramadan fasting on inflammatory biomarkers (IBM) and found that BMI significantly decreased after the month of Ramadan in both the gender.^12^ Kacimi et al. explored that, Ramadan fasting reduce inflammation, risk of developing cancer and improve expectancy of life.^2^ Unalacak et al. also demonstrated that after Ramadan inflammatory markers, including TNF-α, significantly reduced.^10^

Plasma adiponectin levels in our study considerably increased following the Ramadan fasting. Adiponectin hormone level is inversely proportional to body fat mass, hence its level is reduced in obese as compare to normal weight subjects. Bouhlel et al. speculated that elevated adiponectin levels after Ramadan may be due to significant reduction in body fat percentage and body weight.^19^ Our findings are not in agreement with the previous study as Cnop et al. detected that production of some adipokines including adiponectin is correlated to body fat mass.^20^ Few studies have explored adiponectin levels during Ramadan fasting with contrasting findings of either no change,^21^ or decrease in its levels,^22^ as fasting progressed, the amount of adiponectin required for the maintenance of various functions decreased. Studies of Gnanou et al. demonstrated that adiponectin levels decreased then body weight also decreased during the fasting period,^23^ However Ganjali et al. reported non-significantly increased level of adiponectin in obese as well as normal weight individuals.^16^

The studies of Safavi and Rahbar, found no significant changes in adiponectin and TNF-α after Ramadan fasting.^24^ This contrast in result may because of the participants have free access to eat high caloric diet after fasting in the previous study. However, in our study the participants were kept on caloric restriction after fasting and also provided with fibrous oatmeal (White oats). Studies on animal model with caloric restriction following intermittent fasting also showed a significant improvement in adiponectin concentration.^25^

## CONCLUSION

Intermittent Islamic fasting can lower the risk of metabolic syndromes in obese and overweight-weight subjects through reducing weight, BMI, TNF alpha and elevating adiponectin level. Dietary restrictions also showed a beneficial effect on the health. Further studies are required to elucidate the mechanism of improved adiponectin, fall in TNF alpha levels and body weight.

### Authors’ Contribution

All of the Co-authors assisted in data collection.

**RM:** Conceived, designed and did editing of manuscript.

**RM, SK & SA:** Assisted in literature review, manuscript writing and drafting.

**AA:** Did statistical analysis and finalized the write up.
